# Local Variations of Disease

**Published:** 1872-06

**Authors:** 


### Local Variations of Disease.

Among the puzzles in medical science—and there are plenty of them—one of the most interesting and practically useful is the variations of disease in the same locality. Instances of this are numerous, and doubtless familiar to every one, but they are not easily accounted for. In early ages, Rome on her seven hills was a salubrious place, producing men mighty of muscle and a long-lived race, who went forth conquering and to conquer until all the known world was brought a suppliant to her imperial feet. But now a feeble progeny inhabits her site, and the pestilent air of the Campagna poisons the blood of her citizens and visitors.

A century ago Naples and the Madeira Islands were noted as health resorts, and cases of consumption were nigh unknown among the natives. Very different is it now, for foreign visitors and born residents alike succumb to the fatal disease. The latter say it was imported by the outland invalids, but this is a common and easy way to solve a problem which does not really admit any such facile reply.

On the other hand, we can point to many localities in the Mississippi Valley, where practitioners thirty years ago had to combat violent forms of remittent fever and congestive chills, where now such complaints never occur. The same fact characterized the colonial history of the Eastern States. We recollect that a person who had given some attention to antiquarian studies of the kind informed us once that a comparison of the oldest burial memorials of Salem, New Jersey, and other early settlements along the Delaware river, indicates a much greater fatality from miasmatic fevers than now prevails. Some allowance in this class of diseases is to be made for improved treatment—we may say a large allowance—but it does not explain the change in full.

In fact, the "morbid constitution" depends upon a great many factors, and we are far from understanding and appreciating them all as yet. There are some which we hardly more than suspect. Telluric causes we can at least guess at; social ones we can often put our finger upon; but who can say exactly what influences may be exerted by cosmical changes, beyond the reach of chemistry or physics? The idea of some that the progress of the solar system may at times bring us within the maleficient exhalations of bodies foreign to our system, or that certain portions of the earth may suffer from "a pestilent congregation of vapors" of non-telluric origin, is not wholly ridiculous. An attentive study of the changes of the prevalent diseases in definite localities is the first step toward a settlement of such questions.

